# Supplementary material for: Antimicrobial stewardship in remote primary healthcare across northern Australia
Source: PeerJ. 2020 Jul 22;8:e9409. doi: 10.7717/peerj.9409 (PMC7382366; doi:10.7717/peerj.9409)

Supplementary Table 1. Comparison of regional and therapeutic guidelines (Structured Administration and Supply Arrangement (SASA) and Scheduled Substance Treatment Protocols (SSTP) allow RANs and Nurses/Midwives/A&TSIHPs, respectively, to prescribe without telephone order/direction from a medical practitioner).

| **Infection** | **WA – KCPG 2014-16** | **NT – CARPA 2014** | **QLD – PCCM 2016** | **Therapeutic Guidelines v15** |
| --- | --- | --- | --- | --- |
| Impetigo | Benzathine penicillin stat 900mg, OR TMP+SMX: 20+4mg/kg to max 160+800mg BD for 3-5 days, or, 40+8mg/kg to max 320+1600mg daily for 5 days; if pen and sulphonamides allergy use roxithromycin 4mg/kg to max 150mg BD for 5 days.  Do not use topical antibiotics i.e. mupirocin (unless for decolonisation), fusidic acid.  SASA: cephalexin/flucloxacillin | Benzathine penicillin IM 900mg single dose, OR  TMP+SMX: oral 4+20mg/kg/dose BD for 5 days.  Do not use mupirocin due to development of resistance.  SSTP: cephalexin/flucloxacillin/ roxithromycin | TMP+SMX: 8+40mg/kg to max 320+1600mg daily for 5 days, if non-adherence suspected use benzathine penicillin stat;  Different regimens in remote and non-remote areas (BD TMP+SMX only recommended in non-remote areas).  Can use mupirocin 2% intranasal ointment for recurrent staph infections, after sores healed. | Remote sites:  benzathine penicillin 900mg IM single dose, OR  TMP+SMX: 4+20mg/kg to max 160+800mg BD for 3 days, or, 8+40mg/kg to max 320+1600mg daily for 5 days. |
| AOM | With or without perforation:  oral amoxicillin 25mg/kg BD for 7 days, OR azithromycin 30mg/kg single oral dose to 1g max if pen allergy or if associated with trachoma infection in the eye.  At day 7, if no improvement, increase amoxicillin dose to 45mg/kg/dose BD for a further 7 days, or second dose of oral azithromycin increase to 1g.  If a perforation is present and there is persistent discharge despite 7 days of oral antibiotics, add topical ciprofloxacin drops BD for 5 days with planned review.  Recurrent AOM: 25mg/kg/dose BD for 3-6 months, or azithromycin 30mg/kg once a week for 3 months.  SASA: cefaclor for child with acute middle ear infection | Without perforation**:**  Low risk: medical consult – usually no antibiotics.  High-risk (e.g. Indigenous children)**:** oral amoxicillin 25mg/kg/dose, OR TMP+SMX 4+20mg/kg/dose BD for 7 days if pen allergy. If no improvement, increase amoxicillin dose to 50mg/kg/dose BD.  With perforation:  oral amoxicillin 25mg/kg/dose, OR TMP-SMX 4+20mg/kg/dose BD for *14 days* (if pen allergy). At day 7 or 14, if no improvement, increase amoxicillin dose to 50mg/kg/dose BD and *add ciprofloxacin drops* (2-5 drops 2-4 times a day)  Recurrent AOM: amoxicillin 25mg/kg/dose BD for 2 months.  SSTP: azithromycin | Indigenous without perforation: amoxicillin (adult: 500mg tds for 7 days, child: 50 mg/kg/dose BD for 7 days to 1g max BD). At day 7, if no improvement, increase amoxicillin dose to 500 mg tds (adult), 90 mg/kg/dose BD to 1g max BD (child) for further 7 days.  Indigenous with perforation: amoxicillin (adult: 500 mg tds, child: 90 mg/kg/dose BD to 1g max BD for 14 days.  Pen allergy: azithromycin (if older than 6 months) 30 mg/kg to 500mg stat max, OR TMP+SMX 160/800 mg (4+20mg/kg) BD for 5 days.  Non-Indigenous and unwell: amoxicillin 500 mg tds (child: 30mg/kg/dose BD to 1g BD max) for 5 days, OR TMP+SMX (4+20mg/kg) 160/800mg BD for 5 days (if allergy).  If discharge present for >14 days, add 0.3% ciprofloxacin ear drops BD. | Amoxicillin 15mg/kg to 500mg q8h max for 5 days, or, 30mg/kg to 1g BD for 5 days. At day 5, if no improvement, escalate to amox+clav 22.5+3.2mg to 875+125mg BD max for 5-7 days (children: 15+3.75 mg/kg q8h for 5-7 days), and add 0.3% ciprofloxacin ear drops BD for 3 days if discharge present.  Children with pen allergy: cefuroxime 15mg/kg to 500mg max BD for 5 days (child >3 months), OR TMP+SMX 4+20mg/kg to 160+800mg max BD for 5 days (child >1 month) |
| Abscess | Same as for impetigo:  Benzathine penicillin stat 900mg, OR TMP+SMX: 20+4mg/kg to max 160+800mg BD for 3-5 days, or, 40+8mg/kg to max 320+1600mg daily for 5 days; if pen and sulphonamides allergy use roxithromycin 4mg/kg to max 150mg BD for 5 days.  Do not use topical antibiotics i.e. mupirocin (unless for decolonisation), fusidic acid.  SASA: cephalexin/flucloxacillin | Di/flucloxacillin oral 12.5mg/kg/dose QID for 5 days, or, BD for 5 days WITH probenecid oral BD; if pen allergy use clindamycin oral 10mg/kg/dose three times a day for 5 days.  If no improvement, use TMP+SMX oral 4+20mg/kg/dose BD for 5 days.  SSTP: cephalexin/flucloxacillin/ roxithromycin/ phenoxymethylpenicillin/procaine penicillin | Abx as for cellulitis: di/flucloxacillin 500mg (12.5mg/kg) QID for 10 days with review at 3-4 days to potentially cease treatment.  If Group A strep, phenoxymethylpenicillin 500mg (12.5mg/kg) QID for 10 days for 10 days with review at 3-4 days to potentially cease treatment. If poor adherence expected use IM procaine penicillin 1.5g (50mg/kg) for 3 days. If pen allergy, use cephalexin 500mg (12.5mg/kg) QID for 10 days with review at 3-4 days to potentially cease treatment. For pen anaphylaxis, use clindamycin 450mg (10mg/kg) tds for 5-10 days. | Abx for boils/carbuncles:  di/flucloxacillin 500mg (12.5mg/kg) q6h for 5 days, OR  cephalexin 500mg (12.5mg/kg) q6h for 5 days. If pen allergy use clindamycin 450mg (10mg/kg) q8h for 5 days, OR TMP+SMX 160+800mg (4+20mg/kg) q12h for 5 days. |
| Pneumonia | Only for children:  Mild: amoxicillin 35mg/kg per dose (max 500mg) q8h for 5 days, OR if parents/carer not likely to be able to give regular oral medication at home, give procaine penicillin IM 50mg/kg (max 1.5g) for 5 days  Mod/severe: IM benzylpenicillin 30mg/kg (max 3g) q6h while awaiting transfer to hospital.  SASA: cefaclor for child with LRTI, ceftriaxone for severe infections like pneumonia, doxycycline for respiratory tract infection in person >8y/o | Children 2mo to 5yrs:  Mild: procaine penicillin IM once a day for 3 days, OR amoxicillin 25mg/kg/dose TDS for 3 days  Mod/severe: benzylpenicillin IV/IM 30mg/kg/dose QID for 1 day then review.  SSTP: ceftriaxone/doxycycline | Consult Medical Officer/Nurse Practitioner for advice on antibiotic treatment. | Tropical regions, adults:  Mild: amoxicillin 1g oral q8h, OR procaine penicillin 1.5g (or doxycycline or clarithromycin)  Mod: ceftriaxone 2g IV daily OR cefotaxime 2g IV q8h + gentamicin + doxycycline OR clarithromycin – switch to oral amoxicillin + doxy or clarithromycin if improved  Severe: meropenem IV + azithromycin IV (wet season) OR azithromycin IV + ceftriaxone/cefotaxime/pip-taz IV (dry season). |
| STI | If either chlamydia or gonorrhoea is detected always treat for both infections.  If acquired within the Kimberley or other endemic region, use “ZAP pack”:  azithromycin 1g single oral dose + amoxicillin 3g with probenecid 1g single oral dose.  If acquired outside of the Kimberley or outside of other endemic region or area unknown, use “LAC”: azithromycin 1g single oral dose + ceftriaxone single dose 500mg IMI (in 2ml of 1% lignocaine).  If pen allergy: alt. treatment for gonorrhoea ciprofloxacin 500mg single dose, OR azithromycin 2g divided dose. | Chlamydia: azithromycin oral 1g stat  Gonorrhoea: patient AND partner(s) from pen-sensitive area, azithromycin 1g single dose + amoxicillin oral 3g single dose + probenecid oral 1g single dose; patient or partner(s) from pen-resistant area (or unknown partner) and/or is oral/anal gonorrhoea, azithromycin 1g single dose + ceftriaxone IM 500mg single dose (in 2ml of 1% lignocaine)  Trichomonas: metronidazole oral 2g single dose, or, 400mg BD for 7 days if breastfeeding, OR tinidazole oral 2g single dose (not if breastfeeding)  SSTP: ciprofloxacin/doxycycline | Chlamydia/mycoplasma genitalium: azithromycin 1g stat  Gonorrhoea: azithromycin 1g stat + ceftriaxone IM 500mg stat  Trichomonas: metronidazole 2g stat or tinidazole 2g stat (discuss with MO/NP if breastfeeding) | Chlamydia: azithromycin 1g orally, OR doxycycline 100mg q12h for 7 days.  Gonorrhoea (due to AMR, refer to Aus STI guidelines for current recommendations):  Ceftriaxone 500mg IM stat + azithromycin 1g PO stat.  If pen sensitivity confirmed or in pen-sensitive area:  Amoxicillin 3g stat + probenecid 1g stat + azithromycin 1g stat.  Trichomonas: metronidazole 2g stat or tinidazole 2g stat (not safe in pregnancy); if relapse use metronidazole 400mg q12h for 5 days. |
|  | | | | |
| **Infection** | **Difference in recommendation** | | | |
| Impetigo | SXT more prominent in PCCM, in KCPG/CARPA/TG benzathine penicillin encouraged first. PCCM has option for di/flucloxacillin and cephalexin in non-remote community settings. TG recommends BD TMP+SMX dose for 3 days, KCPG/CARPA for 5 days (PCCM only daily dose in remote settings). Mupirocin not recommend for treatment in all guidelines, allowed for decolonisation. TG allows mupirocin in non-remote settings. | | | |
| AOM | CARPA/PCCM recommend stronger/longer dose for AOM with perforation, KCPG no difference between AOMwiP and AOMwoP.  TG recommend amox-clav if no improvement after first line, KCPG/CARPA/PCCM increase dose of amoxicillin and/or azithromycin.  CARPA/TG recommend SXT for penicillin allergy, KCPG azithromycin, PCCM either SXT or azithromycin. KCPG slightly longer duration for rAOM than CARPA. Ciprofloxacin ear drops recommended in all guidelines when discharge present. | | | |
| Abscess | CARPA/PCCM/TG all recommend di/flucloxacillin as first line (also penicillin V/procaine penicillin in PCCM). SXT recommended in CARPA if no improvement. No SXT in PCCM. KCPG group all skin infections (so SXT included). Clindamycin if pen allergy in CARPA/PCCM/TG (roxithromycin in KCPG, and cephalexin also in PCCM). | | | |
| Pneumonia | KCPG & CARPA only has recommendations for young children and are very similar. PCCM doesn’t give recommendation, instead referral. TG has section specific to tropical regions, and has more options for moderate/severe pneumonia, including IV ceftriaxone/gentamicin/doxycycline/clarithromycin/meropenem. | | | |
| STI | All very similar - PCCM routinely recommends ceftriaxone for gonorrhoea and are the only guidelines not to mention treatment options for pen-resistance vs pen-sensitive gonorrhoea. | | | |

Supplementary Table 2. Experiences and feedback from auditors.

| **Kimberley** | **Top End of Northern Territory** | **Far north Queensland** |
| --- | --- | --- |
| 1. *Were data easy to find?* | | |
| - Never before done a report like this  1. IT team set-up data extraction from scratch (now reproducible) 2. Pharmacist went through extracted data to filter records to just those with prescribed anti-infectives 3. Pharmacy assistant entered all data into excel audit spreadsheet  - Clinical coding not routinely used so the indication had to be inferred from the clinical record  1. Records assessed for appropriateness / guideline compliance (some records required referral back to clinical record for additional information) | - A report of all ‘presentations’ to each clinic over the audit period was produced. - This required screening by the auditor as a ‘presentation’ in PCIS often did not match the criteria in the Hot North Project protocol - Screened approx. 1300 events in PCIS to identify approx. 450 events which matched the research criteria (i.e. a presentation which required medical input). - Patient presentations occasionally entered incorrectly (i.e. reason for presentation stated as ‘collect medications’, which may be a routine collection of a blister pack or could include the addition of a new antibiotic prescription on review by the doctor or nurse). - Some antibiotic prescriptions were not recorded in the prescribing area in PCIS (MedChart), occasionally comments at the bottom of the presentation stated antibiotics given but they were never prescribed online. | - Pre-visit: - Patients identified by on-site staff - On-site: - Also used pharmacy book onsite to identify patients, but not always recorded here (e.g. if the printer had broken, nurse forgot etc.) - Bicillins were recorded in a separate book - Some sites had an IV book as well - Post-visit: - Lab results and assessment of appropriateness / guideline compliance were done post-visit - 3 sites also had electronic records – these records revealed more prescriptions that weren’t observed on-site |
| 1. *How long did the audit take?* | | |
| - Data extraction = 4 hours - Identifying records with anti-infectives = 8 hours - Entering data into excel audit spreadsheet = 5 days - Appropriateness / guideline compliance assessment = 5 days - Total ≈ 10.5 days | - 7–15 minutes per episode - >15 if there was an antibiotic, <7 if it was a clinic visit for another reason - Total ≈ 20 days | - On-site data collection = 5–6 hours per site - Post-visit data collection and scoring = 3 weeks - Total ≈ 22.25 days |
| 1. *How variable was* **1.** *and* **2.** *between sites?* | | |
| - No variability – all sites use the same system (MMEX). | - No variability – all sites use the same system | - 2 sites had paper reporting only so patients had to be identified pre-visit |
| 1. *Could the auditors apply consistent definitions?* | | |
| - Confusion around indications like boil/abscess etc. - Without referring back to clinical records, was unclear whether these terms were poor record keeping or something applied by the pharmacy assistant - A true abscess is a surgical condition, so the treatment is surgical, not antibiotics - Some antibiotics were one off prior to immediate transfer to hospital for treatment - Sometimes this could only be established by referring to the clinical record. | - Often difficult to assess severity of conditions without laboratory markers, x-ray reports or detailed clinic notes. - Generally, CARPA definitions were clear – some points for clarification were raised after auditing. | - Often difficult to assess severity of conditions without laboratory markers, x-ray reports or detailed clinic notes |
| 1. *How did auditors group ‘like terms’?* | | |
| - Skin infections: boils, abscess, cellulitis, infected wounds - Ear infections: otitis media with or without discharge - Lower respiratory infections: community acquired pneumonia, infective exacerbation of chronic bronchitis, suppuratives lung disease | - | - |
| 1. *How did auditors deal with ‘un-interpretables’ – how common were they?* | | |
| - Referred back to the original electronic for clarification using further information - If there was insufficient information to make an assessment, recorded it as such rather than guessing - Corresponded with NCAS for specific situations (e.g. how to assess a correct medication for the indication but with unclear information on frequency of dosing) | - There were approx. 10 un-interpretables – sent to the NAPS group for review along with the patient notes and those which could be given a grade were done so by the NAPS team. | - |
| 1. *What were the other major difficulties?* | | |
| - There was only one category relating to compliant with CARPA (or other local guidelines), but there were lots of treatments that were compliant with CARPA but NOT compliant with local guidelines so they had to be marked as complaint. - Had many RHD monthly penicillin injections which would have improved compliance data, while other parts of Australia would have very few - Although attempted to have a different person enter data and assess data, sometimes it was necessary for the assessor to refer to the original clinical records for clarification (which often resulted in a more accurate assessment) | - Assessment of appropriateness always done on a 1 page event summary often with no other PMHx to go off which is fraught - There are a number of areas where the CARPA gives misleading recommendations which when interpreted by untrained staff leads to sub-optimal treatment - NT audit was done before the other two sites and before standardised lists (e.g. of indications) were available | - Given the many sources to refer to, lack of time on-site was a major issue - Many instances of prescribing off guidelines where they are not fit for purpose (particularly SSTIs), making deviation appropriate |
| 1. *How much training did local auditors need?* | | |
| - Pharmacy assistant received training via video-conference - Other auditors (e.g. pharmacist and appropriateness assessor) had no training | - Needed to be trained in the use of PCIS - Needed to be trained in assessing antibiotic prescriptions and be familiar with the NAPS grading system | - The auditors were 2 QSAMS staff familiar with NAPS form as well as a nurse from Thursday Island who also had done a NAPS audit before, so no local auditors were used. |
| 1. *How much phone support did local auditors need?* | | |
| - NCAS team contacted via email for assistance on half a dozen records (e.g. how to assess compliance) | - Needed support from remote health to identify which prescribers were locum staff vs permanent staff - Needed support from the NAPS team for assessing some prescriptions - Needed support from PCIS to run the reports necessary to identify patients | - NCAS contacted approx. 5 times via phone to ensure interpretation of scoring was consistent |
| 1. *Would they do this again?* | | |
| - Probably not - With some support, this could have been done by a senior medical student as part of a project. | - No | - |
| 1. *What might encourage or discourage them from doing this again?* | | |
| - Huge amount of man-power needed - However, results were very informative - Subsequent audits would be easier since data extraction / report template are established | - Too repetitive and time-consuming - Tool itself was pretty good | - Extremely time consuming and expensive - Will be easier once electronic prescribing and charting are consistent - Data beneficial for health service planning |
| 1. *Any suggestions for improvement?* | | |
| - Perhaps add separate category: “not complaint with local guidelines but compliant with national guidelines” - Difficult to grade appropriateness/guideline compliance of combination prescriptions (e.g. flucloxacillin & probenecid) where dose of one is adequate but not the other | - Recommend to update NAPS indication list to better reflect common primary health presentations | - Provide primary health common presentations examples in training for consistent scoring across jurisdictions - Add section to distinguish medical onsite review vs medical offsite review (phone order) |
| 1. *Are there opportunities for improved integration / data collection in relation to envisaged systems improvements?* | | |
| - Confusing guidelines for treating suspected worms (e.g. stat dose of albendazole for 3 days for whipworm and Strongyloidiasis but 1 day for others), CARPA guideline for routine worming is stat dose before and after wet - Confusion in CARPA: e.g. treating diarrhoea of >7 days = test stool but unclear whether to give empirical metronidazole or await result (pg174) - CARPA routinely advises to give antibiotics if in doubt, rather than following stewardship principles - Issues with MMEX system: - Reverts to default options of PRN or one original pack of medication - Human entry errors (e.g. 30 worming tablets when almost certainly would’ve been 3) - Some prescriptions likely given for more than one family member which would explain mismatch in quantities (e.g. 12 albendazole tablets) - Audit revealed that more training is needed on how to use MMEX in conjunction with the KAMS medication policy to remote health centre staff - The relative newness of the policy, and the turnover of staff / use of agency staff meant that some staff were not using MMEX appropriately - Weekly training (i.e. clinical scenarios) is helping to improve this | - Currently implementing new IT system for all of NT health – opportunity to include mandatory reporting of indication (included in requests to vendor) | - Electronic systems being rolled-out state-wide, so no more paper-based sites (however, not all sites will have the same IT system) - Changes to RIPRN model for prescribing currently unknown – unsure about impact to PCCM. - Electronic prescribing should record all medications used for a patient – not just those dispensed (i.e. stat IM, IVAB given whilst in clinic). - Make AOM treatment flowchart easier to access and follow - Should there be a blanket Bactrim rule in the top end for SSTI? |


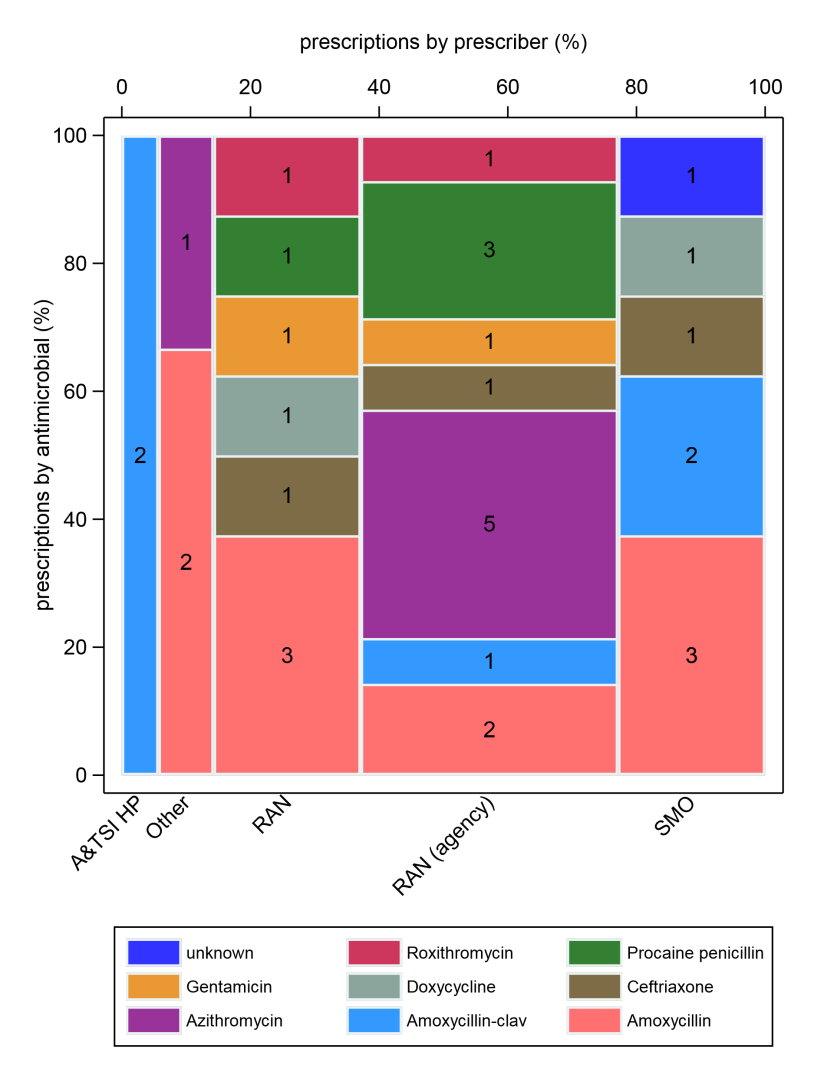
Supplementary Figure 1. Antimicrobial use for LRTI in the Top End of the NT by health professional and antimicrobial. Numbers in middle of columns are the number (not percentage) of antimicrobials used for that health professional/antimicrobial group.

A&TSI HP: Aboriginal and Torres Strait Islander health practitioner; RAN: remote area nurse; SMO: staff medical officer. Note: Ceftriaxone & Gentamicin = IV, all others = PO


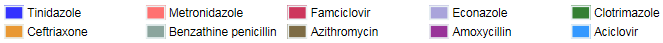

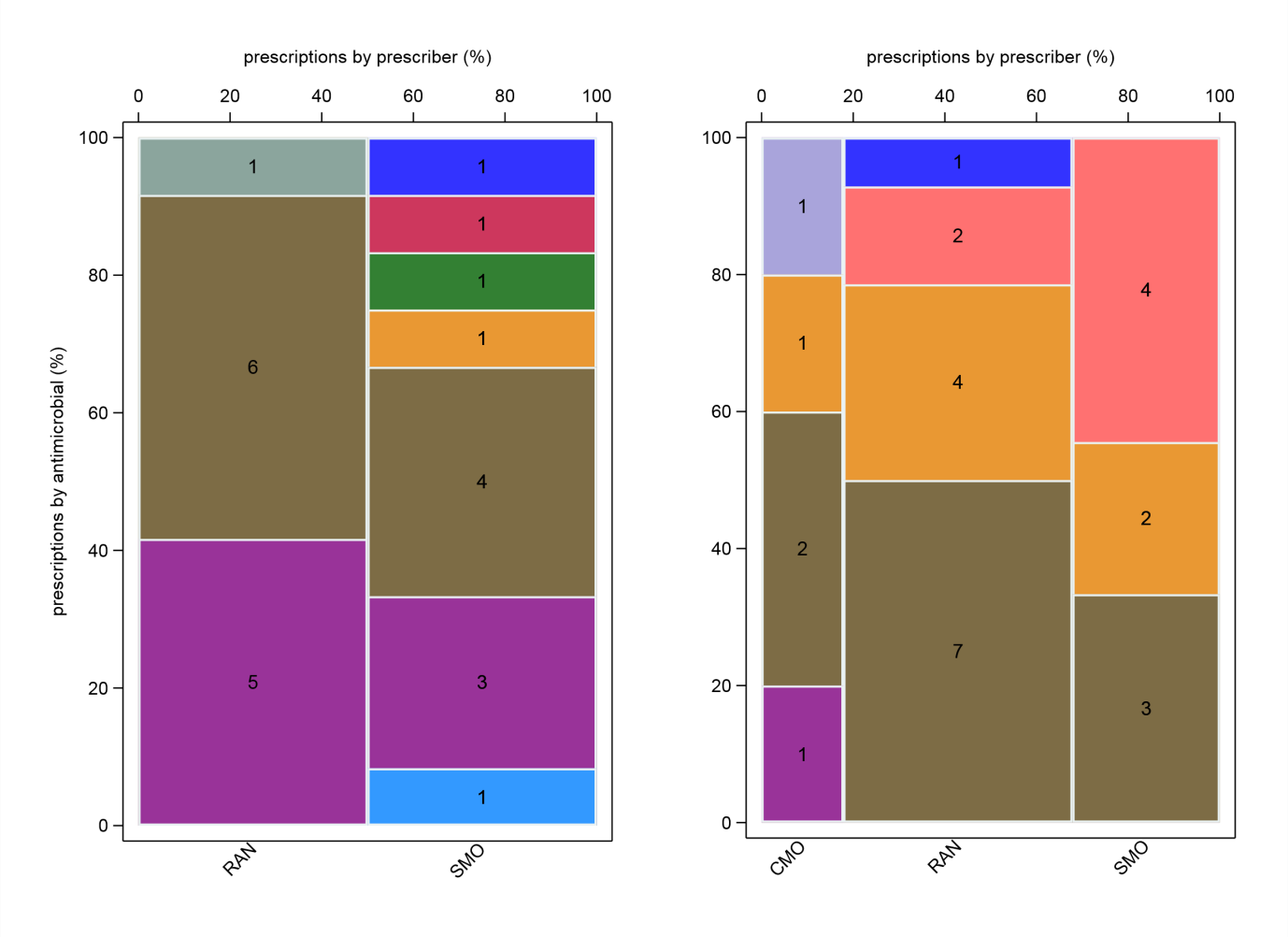
Supplementary Figure 2. Antimicrobial use for STI in the Kimberley (left) & far north Queensland (right) by prescriber and antimicrobial. Numbers in middle of columns are the number (not percentage) of antimicrobials used for that health professional/antimicrobial group.

RAN: remote area nurse; SMO: staff medical officer; CMO: contracted medical officer.

Note: Kimberley – Aciclovir & clotrimazole = topical, Ceftriaxone = IV/IM, all others = PO (8 of the Azithromycin prescriptions in WA were “ZAP” packs (azithromycin/amoxicillin combination)). Far north Queensland – Econazole = topical, Ceftriaxone = 6 IM / 1 IV (by a RAN), all others = PO

Supplementary Table 3: Appropriateness of antimicrobial use by health professional and syndrome in each jurisdiction

|  |  | EI | GI | LRTI | RHD | SSTI | STI | URTI | UTI | Other | Total |
| --- | --- | --- | --- | --- | --- | --- | --- | --- | --- | --- | --- |
| Aboriginal & Torres Strait Islander Health Practitioner | | -;  -;  - | -;  -;  - | -;  0/2;  - | 1/1;  -;  - | -;  -;  - | -;  -;  - | -;  1/1:  - | -;  -;  - | -;  -;  - | 1/1;  1/3;  - |
| Nurses | Registered nurse | 1/1; 2/2;  - | -;  6/6;  - | -;  1/1;  - | -;  -;  - | 1/1; 4/5; 5/7 | -;  -;  - | -;  -;  - | -;  -;  - | 0/1;  -;  2/3 | 2/3; 13/14; 7/10 |
|  | Remote area nurse | 6/7; 5/6;  - | 9/9; 5/5; 6/6 | 1/1; 7/8; 0/1 | 2/2; 13/13; - | 38/41; 10/13; 12/20 | 12/12; -; 12/14 | 3/6; 1/3; 4/4 | 2/2; 1/1; 1/1 | 16/16; 2/2; 5/5 | 89/96; 44/51; 40/51 |
|  | Remote area nurse (agency) | -;  2/2;  - | -;  3/4;  - | -; 12/14; - | -;  5/5;  - | -; 21/27; - | -;  1/1;  - | -;  7/7;  - | -;  9/9;  - | -;  5/7;  - | -;  65/76;  - |
|  | Total | 7/8; 9/10; - | 9/9; 14/15; 6/6 | 1/1; 20/23; 0/1 | 2/2; 18/18; - | 39/42; 35/45; 17/27 | 12/12; 1/1; 12/14 | 3/6; 8/10; 4/4 | 2/2; 10/10; 1/1 | 16/17; 7/9; 7/8 | 90/99; 122/141; 47/61 |
| Doctors | Staff medical officer | 8/9;  -;  1/8 | 16/17; 3/4; 7/8 | 18/18; 5/7; 1/6 | 3/3; 3/4; 11/12 | 33/37; 4/7; 29/46 | 12/12; 5/5; 9/9 | 1/2;  -;  9/12 | 12/13; 2/3; 2/3 | 10/13; 3/5; 8/16 | 113/124; 25/35; 77/120 |
|  | Contracted medical officer* | -;  -;  0/2 | -;  -;  4/5 | -;  -;  0/1 | -;  -;  2/2 | -;  -;  6/17 | -;  -;  4/5 | -;  -;  - | -;  -;  2/2 | -;  -;  3/4 | -;  -;  21/38 |
|  | Other | -;  3/3; 1/1 | -;  -;  - | -;  1/2;  - | -;  -;  - | 0/1; 4/5;  - | -;  -;  - | -;  1/1;  - | -;  -;  - | -;  2/3; 1/6 | 0/1; 11/14;  2/7 |
|  | Total | 8/9; 3/3; 2/11 | 16/17; 3/4; 11/13 | 18/18; 6/9; 1/7 | 3/3; 3/4; 13/14 | 33/38; 8/12; 35/63 | 12/12; 5/5; 13/14 | 1/2; 1/1; 9/12 | 12/13; 2/3; 4/5 | 10/13; 5/8; 12/26 | 113/125; 36/49; 100/165 |
| Total | | 15/17; 12/13; 2/11 | 25/26; 17/19; 17/19 | 19/19; 26/34; 1/8 | 6/6; 21/22; 13/14 | 72/80; 43/57; 52/90 | 24/24; 6/6; 25/28 | 4/8; 10/12; 13/16 | 14/15; 12/13; 5/6 | 26/30; 12/17; 19/34 | 205/225; 159/193; 147/226 |

Notes: values in cells represent the fraction of antimicrobials used that were appropriate (WA; NT; QLD). >75% appropriate in green, <60% appropriate in red for groups with denominators ≥20. Appropriateness not assessable – WA: 6 (plus 12 unknown prescriber); NT: 3; QLD: 3.

EI – ear infection, GI – gastrointestinal infection, LRTI – lower respiratory tract infection, RHD – rheumatic heart disease, SSTI – skin & soft tissue infection, STI – sexually transmitted infection, URTI – upper respiratory tract infection, UTI – urinary tract infection.

* CMOs only in one of the five Queensland clinics

Supplementary Table 4. Frequency of antimicrobial use by health professional (disaggregated), antimicrobial (grouped), appropriateness of antimicrobial use, issues with antimicrobial use and guideline compliance.

|  | **WA** | | **NT** | | **QLD** | | **Total** | |
| --- | --- | --- | --- | --- | --- | --- | --- | --- |
|  | No. | Col% | No. | Col% | No. | Col% | No. | Col% |
| **Health professional** |  |  |  |  |  |  |  |  |
| A&TSI health worker | 1 | <1% | 3 | 2% | 0 | 0% | 4 | 1% |
| Casual rural practitioner | 0 | 0% | 9 | 5% | 0 | 0% | 9 | 1% |
| Contracted medical officer | 0 | 0% | 0 | 0% | 39 | 17% | 39 | 6% |
| Dentist | 0 | 0% | 2 | 1% | 0 | 0% | 2 | <1% |
| Locum medical officer | 1 | <1% | 0 | 0% | 0 | 0% | 1 | <1% |
| Registered nurse | 3 | 1% | 14 | 7% | 10 | 4% | 27 | 4% |
| Remote area nurse | 97 | 40% | 52 | 27% | 53 | 23% | 202 | 30% |
| Remote area nurse (agency) | 0 | 0% | 76 | 39% | 0 | 0% | 76 | 11% |
| Staff medical officer | 129 | 53% | 37 | 19% | 120 | 52% | 286 | 43% |
| Unknown | 12 | 5% | 0 | 0% | 0 | 0% | 12 | 2% |
| Visiting specialist | 0 | 0% | 3 | 2% | 7 | 3% | 10 | 1% |
| Total | 243 | 100% | 196 | 100% | 229 | 100% | 668 | 100% |
| **Antimicrobial (grouped)** |  |  |  |  |  |  |  |  |
| Antifungal | 17 | 7% | 10 | 5% | 17 | 7% | 44 | 7% |
| Antiparasitic | 38 | 16% | 29 | 15% | 18 | 8% | 85 | 13% |
| Antiviral | 2 | 1% | 1 | 1% | 0 | 0% | 3 | <1% |
| Antibiotic | 186 | 76% | 155 | 79% | 194 | 85% | 537 | 80% |
| Unknown | 0 | 0% | 1 | 1% | 0 | 0% | 1 | <1% |
| Total | 243 | 100% | 196 | 100% | 229 | 100% | 668 | 100% |
| **Appropriateness** |  |  |  |  |  |  |  |  |
| Optimal | 145 | 60% | 137 | 70% | 120 | 52% | 402 | 60% |
| Adequate | 70 | 29% | 22 | 11% | 27 | 12% | 119 | 18% |
| Suboptimal | 12 | 5% | 22 | 11% | 20 | 9% | 54 | 8% |
| Inadequate | 10 | 4% | 12 | 6% | 59 | 26% | 81 | 12% |
| Not assessable | 6 | 2% | 3 | 2% | 3 | 1% | 12 | 2% |
| Total | 243 | 100% | 196 | 100% | 229 | 100% | 668 | 100% |
| **Issues with antimicrobial use*** |  |  |  |  |  |  |  |  |
| Allergy mismatch | 0 | 0% | 1 | 1% | 1 | <1% | 2 | <1% |
| Micro mismatch | 3 | 1% | 0 | 0% | 2 | 1% | 5 | 1% |
| Indication not required | 10 | 4% | 13 | 7% | 22 | 10% | 45 | 7% |
| Incorrect route | 0 | 0% | 0 | 0% | 6 | 3% | 6 | 1% |
| Incorrect dose / frequency | 18 | 8% | 14 | 7% | 29 | 13% | 61 | 9% |
| Duration too long | 16 | 7% | 3 | 2% | 9 | 4% | 28 | 4% |
| Duration too short | 7 | 3% | 0 | 0% | 12 | 5% | 19 | 3% |
| Spectrum too broad | 5 | 2% | 12 | 6% | 23 | 10% | 40 | 6% |
| Spectrum too narrow | 4 | 2% | 7 | 4% | 22 | 10% | 33 | 5% |
| **Guideline compliance** |  |  |  |  |  |  |  |  |
| Compliant with local guidelines | 170 | 70% | 130 | 66% | 36 | 16% | 336 | 50% |
| Compliant with TG | 30 | 12% | 11 | 6% | 58 | 25% | 99 | 15% |
| Non-compliant | 21 | 9% | 35 | 18% | 99 | 43% | 155 | 23% |
| Directed therapy | 13 | 5% | 8 | 4% | 30 | 13% | 51 | 8% |
| No guidelines available | 3 | 1% | 9 | 5% | 3 | 1% | 15 | 2% |
| Not assessable | 6 | 2% | 3 | 2% | 3 | 1% | 12 | 2% |
| Total | 243 | 100% | 196 | 100% | 229 | 100% | 668 | 100% |

Note: WA: Kimberley region of Western Australia; NT: Top End of the Northern Territory; QLD: far north Queensland

*Denominator is the total number of assessable antimicrobials used (WA: 237, NT: 193, QLD: 226, Total: 656); there can be more than one issue with a single antimicrobial; treatment may have been deemed appropriate even if one (or more) of these issues were ‘true’

Appendix A. Data collection form.

Appendix B. Algorithm to assess appropriateness of antimicrobial used.


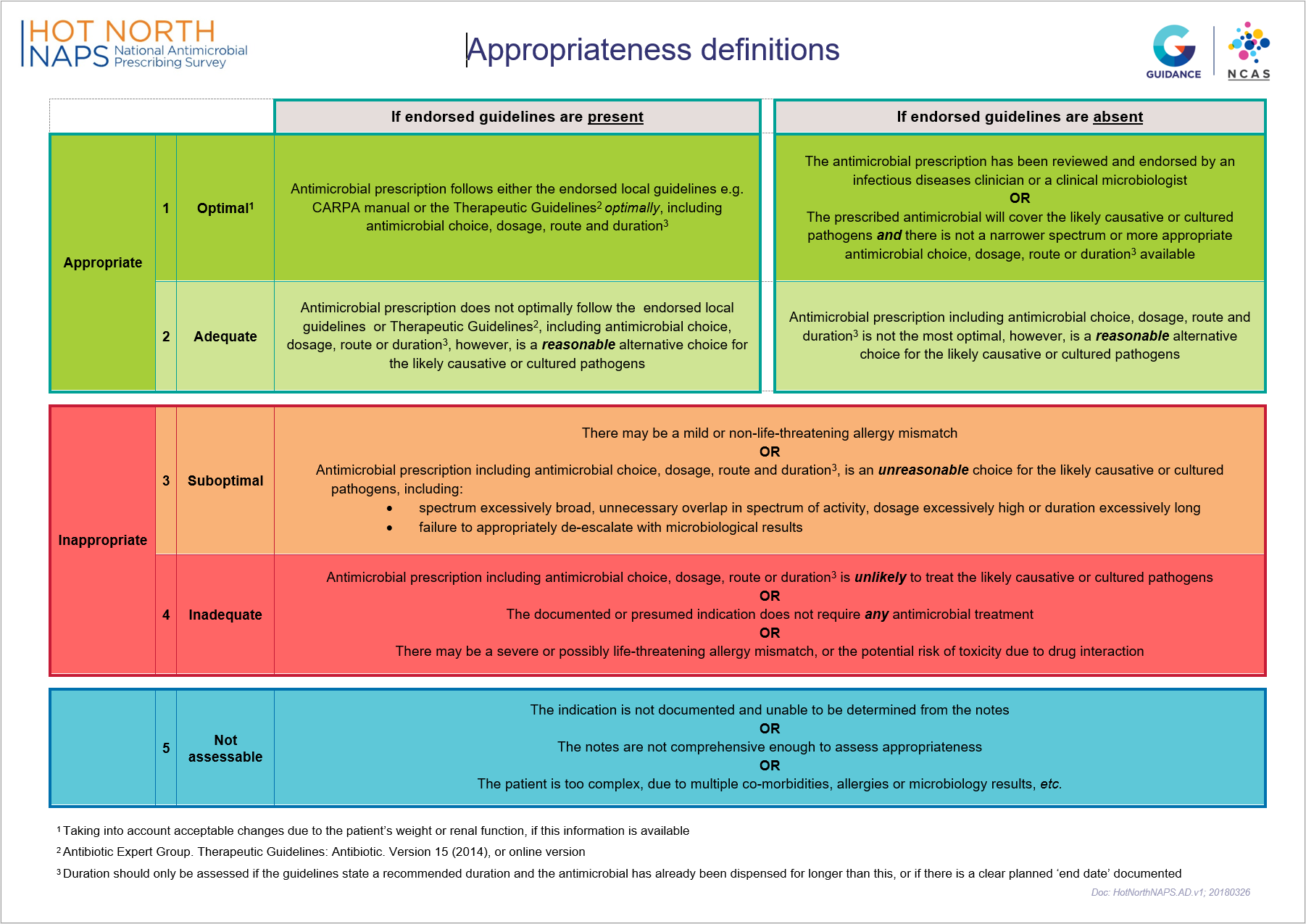

Supplement: Supplemental Information 1 [file peerj-08-9409-s001.docx]
